# Metabolomics combined with transcriptomics reveals the formation mechanism of different leaf colors of *Heuchera micrantha*

**DOI:** 10.3389/fpls.2025.1672924

**Published:** 2025-09-26

**Authors:** Yuxi Wang, Yumeng Tang, Xi Chen, Xiaodong Yang, Qi Zhou, Yueheng Hu, Xiaohua Meng, Jialin Peng

**Affiliations:** ^1^ College of Environmental Ecology, Jiangsu Open University, Nanjing, China; ^2^ College of Horticulture, Nanjing Agricultural University, Nanjing, China

**Keywords:** *Heuchera micrantha*, color-leafed plants, metabolomics, transcriptomics, flavonoid metabolism

## Abstract

**Introduction:**

The vivid colors of color-leafed plants endow plants with unique ornamental value. At present, there are many researches focusing on the mechanism of flower color formation, while there is less interesting research on color-leafed plants. As an excellent color-leafed plant, *Heuchera micrantha* has only been studied for its pigment content and physiological characteristics, and the mechanism of color-leafed formation has not been characterized yet.

**Methods:**

In this study, we used two varieties of *Heuchera micrantha* with green and red leaves as materials, and employed a combination of metabolomics and transcriptomics to reveal the molecular mechanisms underlying the formation of different color leaves.

**Results:**

Through observation of phenotype, analysis of metabolomics and transcriptomics, and combined analysis of multi-omics, it was found that differential metabolites and differentially expressed genes were enriched in flavonoid metabolism and related pathways. Nine MYB and bHLH transcription-factor candidates implicated in flavonoid metabolism were selected and functionally annotated; five are predicted to act as activators and four as repressors of the flavonoid biosynthetic pathway.

**Discussion:**

In summary, this study provides important insights into the coloring mechanism of color-leafed plants and provides gene reserves for subsequent targeted breeding.

## Introduction

The ornamental value of ornamental plants largely comes from their colorful appearance. The color of plants mainly comes from pigments such as chlorophyll, carotenoids, flavonoids, betaine, etc (Tanaka et al., 2008; Tanaka et al., 2009). These pigments not only make plants look beautiful, but also help them carry out photosynthesis, attract pollinators, and spread seeds (Cazzonelli, 2011; Wen et al., 2020; Singh et al., 2021; Tanaka, 2025). In addition, they also help plants resist external pressures such as oxidation, ultraviolet radiation, and microbial pathogens (Imène et al., 2010; Cazzonelli, 2011; Kristanto et al., 2021).

As one of the important ornamental features, the study of the mechanism of plant color formation has a long history. Research has shown that changes in the color of plants are caused by variations in the types and contents of their intrinsic plant pigments. Among them, flavonoids are the most widely present type of plant pigments. It can endow plants with a wide range of colors and determine the color of most angiosperms (Tanaka et al., 2008; Zhao and Tao, 2015; Yonekura-Sakakibara et al., 2019). Numerous studies have shown that the flavonoid metabolism pathway is highly conserved during plant evolution (Winkel Shirley, 2001; Petroni and Tonelli, 2011; Wen et al., 2020), and its biosynthesis is regulated by flavonoid biosynthesis structural genes and upstream related transcription factors, such as myeloblastosis (MYB), basic helix-loop-helix (bHLH), WRKY, YABBY, WD40-repeat proteins (WD40) (Koes et al., 2005; Pang et al., 2009; Cong et al., 2021; Kayani et al., 2021).

There are many colorful organs in ornamental plants, including flowers, fruits, leaves, and stems. However, current research on the coloring mechanism and regulation of ornamental plants is mostly focused on flower colors (Yin et al., 2021; Erickson and Pessoa, 2022; Shen et al., 2024), with few research on colored leaves. Color-leafed ornamentals can maintain their vibrant colors throughout the entire growing season, prolonging viewing time with minimal maintenance costs and adding a sense of layering to urban landscapes. Therefore, it is of great significance to conduct research on the coloring mechanism and regulation of colorleafed plants. This can help explore and understand the diversity of plant color-leafed, as well as screening candidate genes for color-leafed regulation. It also helps to selectively cultivate more color-leafed plants to beautify the environment. *Heuchera micrantha* is a highly valuable horticultural ornamental plant known for its unique leaf shape and colorful leaves (Rabe and Soltis, 1999). Its leaves exhibit a diverse range of colors, including green, yellow, red, and multiple colors, and its color changes to varying degrees with light, temperature, and other environmental factors. Meanwhile, *Heuchera micrantha* exhibits strong environmental adaptability. It has resistant to cold and can withstand lower temperatures (Wang and Deng, 2014). At present, research on the mechanism and regulation of plant color formation is mainly focused on flower color or fruit color (Erika Cavallini and Finezzo, 2015; Yin et al., 2021; Erickson and Pessoa, 2022). Research on *Heuchera micrantha* involves its physiological characteristics (Wang and Deng, 2014), lipid metabolism (Gong et al., 2023), alkaloid metabolism (Gong et al., 2024), flavonoid species (Wilkins and Bohm, 1976), etc., while there is a lack of research on the formation and regulatory mechanisms of its different leaf colors.

In this study, we have taken *Heuchera micrantha* with different leaf colors asresearch object, and use transcriptome and metabolome to comprehensively understand the activity level and key nodes of metabolic pathways. It uncovers the pathways and genes driving the formation of color-leafed in *Heuchera micrantha* and elucidates how color-leafed forms. It also provides potential genetic resources and theoretical support for the genetic improvement of color-leafed traits in horticultural plants and the cultivation of new varieties.

## Methods

### Cultivation conditions for plant materials

We have chosen leaves that are representative and of pure color of *Heuchera micrantha*, namely red and green. The plants were planted in a greenhouse with a light exposure time of 16 hours at a temperature of 24°C, a dark time of 8 hours at a temperature of 16°C, and a humidity of 70% throughout the entire process. At least 5 biological replicates of each color of leaf were collected at the same leaf age of approximately ten leaves, with a portion used for subsequent color measurements and another portion frozen at -80°C for future experiments, such as metabolic analysis and transciptomic analysis.

### Measurement of color-leafed phenotype

The Minolta CR-400 handheld colorimeter (Konica Minolta, Japan) were used to, ascertain the color attributes of two different colored *Heuchera micrantha* leaves, referring to the CIELab color system (Chen et al., 2013). Based on the instruction manual, the measured values of luminance (L*) and chromatic elements a* and b* were obtained, and then the saturation or chromaticity (C*) was calculated using the formula C *=(a*^2^+b*^2^)^1/2^ (Gonnet, 1998). The experiment was conducted six times and the average was taken to ensure reliability.

### Metabolomics analysis

Each sample contains six biological replicates for metabolomics analysis. Metabolite determination were conducted by LC Biotechnology Co., Ltd. (Hangzhou, China). The sample metabolites of leaf sample were extracted with 80% methanol, followed by Ultra Performance Liquid Chromatography–Tandem Mass Spectrometry (UPLC-MS/MS) analysis using the Vanquish Flex Ultra-high Performance Liquid Chromatography system (Thermo Fisher Scientific, Germany) and Q-Exactive Plus system (Thermo Fisher Scientific, Germany). XCMS software is used to preprocess the collected mass spectrometry data, including peak picking, peak grouping, retention time correction, secondary peak grouping, isotope and adduct labeling (Smith et al., 2006). Data analysis was performed using the R package (**Supplementary Table S2**), which included statistical analysis such as normalization, hierarchical clustering, PCA, and PLSDA. Significant metabolites were identified through a combination of P-values, fold changes, and VIP scores. GSEA and MSigDB enrichment analysis pinpointed differential KEGG gene sets, with significance determined by NES, p-values, and q-values. Metabolites were ultimately mapped to their respective pathways.

### Transcriptome construction and analysis of differential expression genes

Total RNA was extracted from different colored leaves using RNAiso Reagent (Takara, Tokyo, Japan) according to the manufacturer’s instructions, six cDNA libraries for Green and Red leaves were constructed with three biological replicates for each samples. The RNA libraries were sequenced on the illumina NovaseqTM 6000 platform by LC Bio Technology CO.,Ltd (Hangzhou, China). Subsequent bioinformatic analysis was performed using the OmicStudio tools at https://www.omicstudio.cn/tool (Lyu et al., 2023).

### Joint analysis of metabolomics and transcriptomics

Significant differentially expressed genes and metabolites are screened in transcriptome and metabolome data, respectively. Then, based on the intersection of these pathways, differential expressed genes and differential metabolites within the intersecting pathways are sought. Subsequently, the structure is depicted as a network diagram, displaying the associated data, with a particular emphasis on the pathways of interest and the differential expression patterns of RNAs. The R package used for data analysis is shown in the **Supplementary Table S2**.

### Statistical analysis

Data are shown as means. Student’s t-test was used for comparisons between 2 groups. Differentially expressed genes are classified based on FC (Fold Change) ≥2 or FC ≤ 0.5 (i.e. absolute value of log_2_FC≥1) and False Discovery Rate (FDR) value<0.05 (|log_2_FC|≥1 & FDR<0.05) as the standard. Differential metabolites need to satisfy: FC≥1.2 or FC≥1/1.2 (p value < 0.05、VIP ≥1). In the heat map drawing, we use Zero Mean Unit Variance Normalization to normalize each row of data, and the formula is: Z=(X-μ)/σ (Paulson, 1942).

## Results

### Analysis of color-leafed phenotype

**Figure 1:**
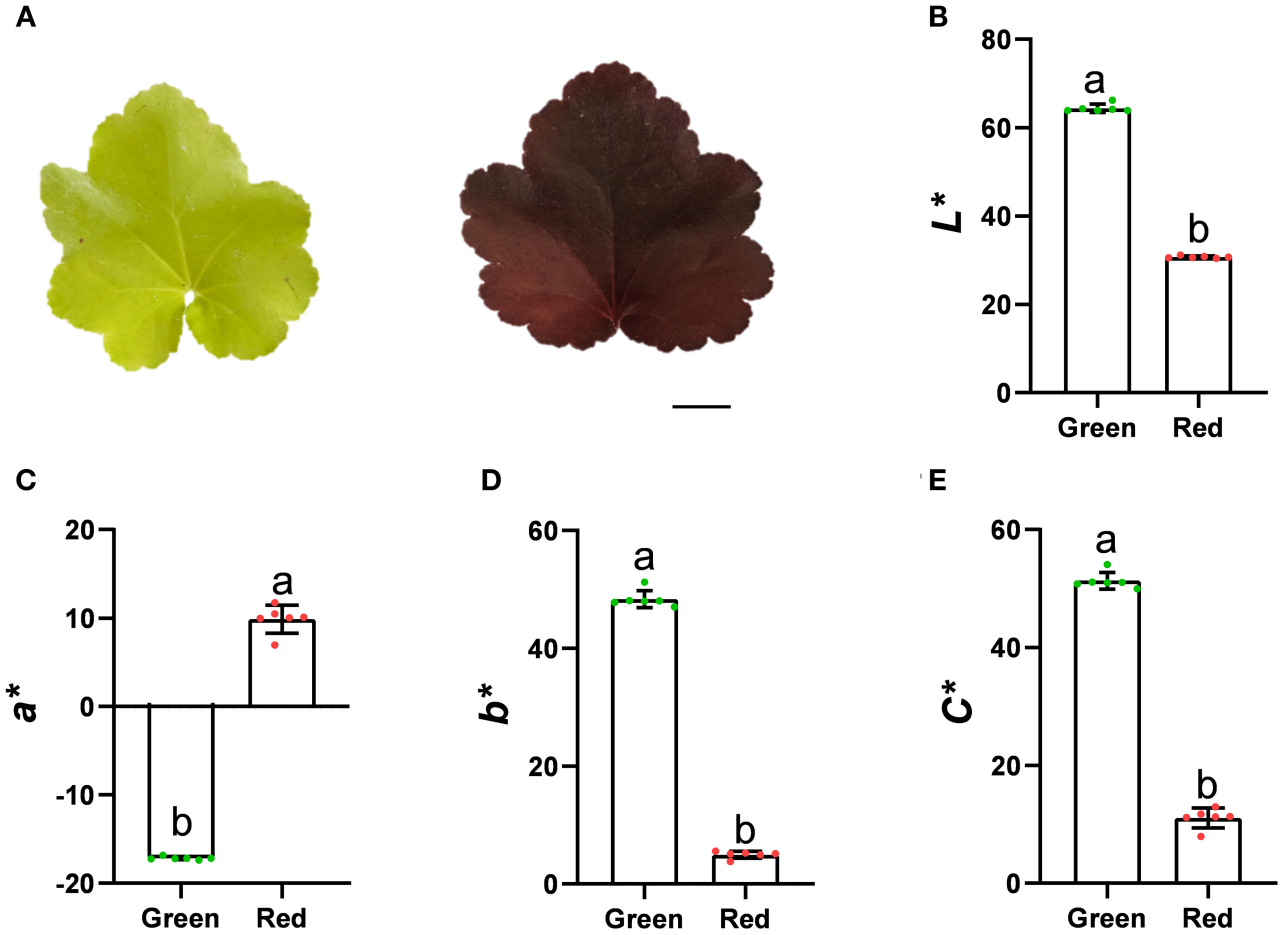
The phenotype of *Heuchera micrantha* leaves with different colors. Bars = 1 cm. **(A)** *Heuchera micrantha* with different leaf colors, namely Green and Red. **(B-E)**. CIELab* color parameters of leaves of three different colors, including values of lightness (L*), a*, b*, and chroma (C*). Error bars indicate the standard deviation of six biological replicates.

Under the same growth state, *Heuchera micrantha* with different leaf colors, namely green and red, were selected as the plant materials for this study (**Figure 1A**). In order to quantify its leaf color phenotype, we used the Minolta CR-400 handheld colorimeter to measure the L*, a*, b* value and calculate the C value of different leaves. The results indicate that the brightness (L*) of green leaves is significantly higher than that of red leaves (**Figure 1B**). In the measurement of a* value, green leaves are negative, indicating that they belong to the green system, while red leaves are positive, indicating that they belong to the red system, which corresponds exactly to the range of a* channel changes from red to green (**Figure 1C**). The b* values of both are positive, indicating that they belong to the yellow category, and the value of green leaves is significantly higher than that of red leaves (**Figure 1D**). Furthermore, the C* value of the two-color leaves were calculated and compared, and found that the chorma of green leaves was significantly higher than that of red (**Figure 1E**).

### Metabolomic profiling of leaves with different colors and identification of metabolic pathways

**Figure 2:**
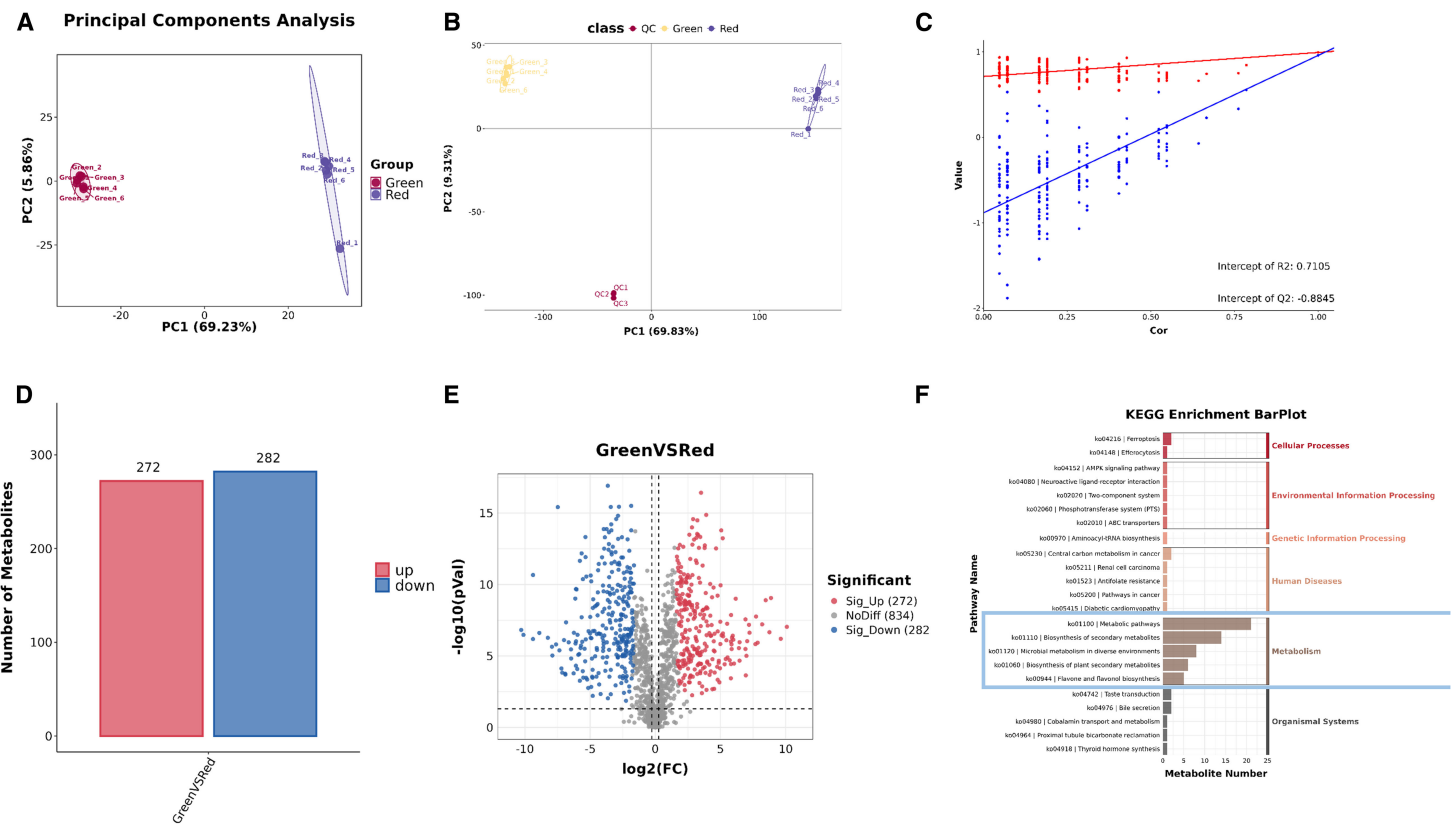
Overview of the metabolite profile of *Heuchera micrantha* leaves with different colors. **(A)** The principal component analysis (PCA) diagram illustrates sample separation based on leaf color categories. **(B, C)** The combined analysis of PCA plots and scatter plots shows that good separation of data between different groups and demonstrates the predictive accuracy of the model. **(D, E)** The combined analysis of bar chart and volcano chart indicates that the number of metabolites identified in each leaf color class. **(F)** The KEGG hierarchical bar chart of differential metabolites.

UPLC-MS/MS analysis was performed to quantify the total metabolite profiling, in order to further explore the main metabolic changes between the “Green” and “Red” leaves. Principal Component Analysis (PCA) showed that PC1 is 69.23% and PC2 is 5.86% (**Figure 2A**). Moreover, statistical analysis was conducted on the metabolome using Partial Least Squares Discriminant Analysis (PLS-DA), as shown in **Figures 2B, C**. The PLS-DA score plot shows that PC1 is 69.83% and PC2 is 9.31%. Each point on the plot corresponds to a sample, demonstrating clear aggregation within the sample groups and distinct dispersion between the groups. This pattern strongly suggests that the “Green” and “Red” leaves exhibit different metabolic expression profiles (**Figure 2B**). The permutation test chart demonstrates that the metabolomic model exhibits no signs of overfitting (**Figure 2C**).

Subsequently, a total of 29836 primary metabolites and 2351 secondary metabolites were identified. Aanalysis was focused on secondary metabolites. In the “Green” group, 272 secondary metabolites were significantly higher than those in the “Red” group, 282 secondary metabolites were significantly lower, and 834 secondary metabolites showed no significant difference between the two groups (**Figures 2D, E**). For Kyoto Encyclopedia of Genes and Genomes(KEGG) enrichment analysis of differential metabolites, the KEGG hierarchical bar chart shows that the KEGG primary classification of differential metabolites mainly focuses on changes in “Metabolic”, as shown in the blue box in **Figure 2F**.

### Analysis of key metabolic pathways and key differential metabolites

**Figure 3:**
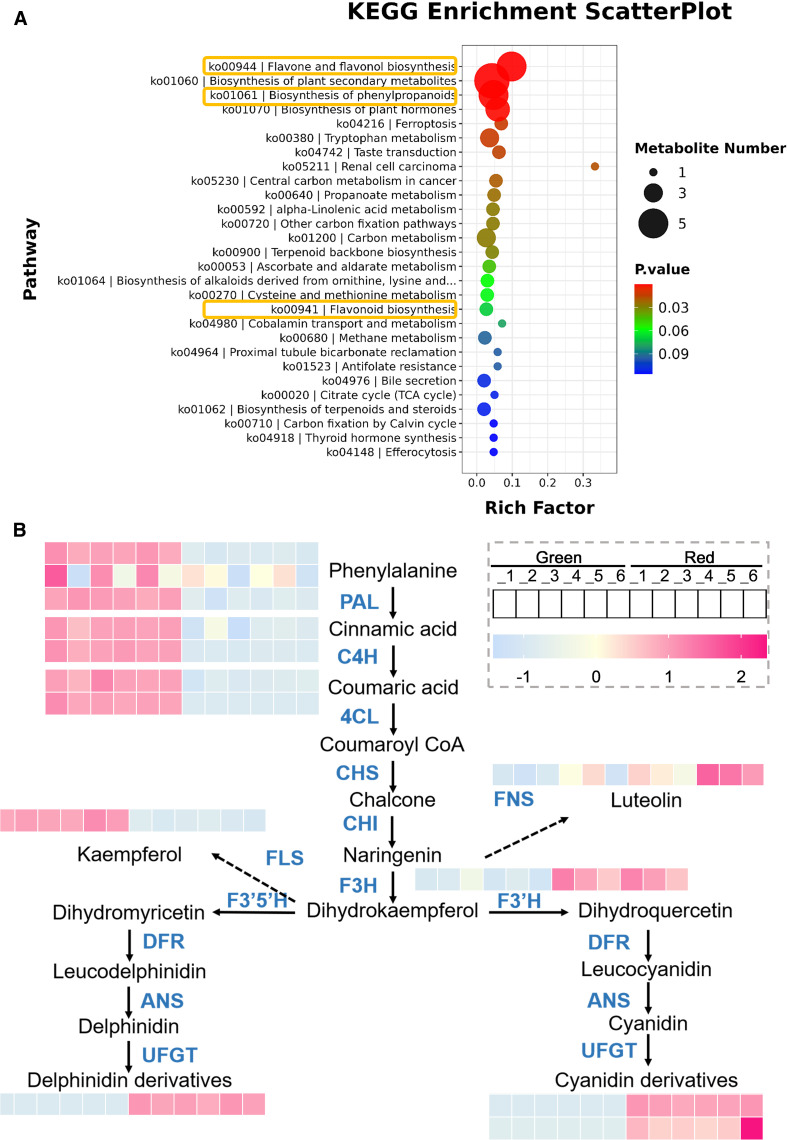
Analysis of key metabolic pathways. **(A)** KEGG enrichment bubble plots. The size of the dots represents the number of metabolites involved in each pathway, with larger dots indicating higher quantities. The color gradient from blue to red represents the P-value, with red indicating a more significant enrichment. **(B)** Metabolic pathway diagram. The arrow indicates the direction of metabolic flow, and the color gradient from blue to orange represents the quantity of metabolites, with pink indicating a higher quantity.

Based on the previous results, differential metabolites were mainly enriched in metabolic pathways. To further investigate their key pathways, we generated KEGG enrichment bubble plots. As shown in **Figure 3A**, the pathways of differential metabolite enrichment include those related to flavonoid metabolism.

In order to understand the trend of metabolite changes, we plotted a differential metabolite metabolic pathway map (**Figure 3B**). The results showed that in the early stages of metabolism, including phenylalanine, cinnamic acid, and coumaric acid, the content in “Green” group was significantly higher than that in “Red”, which may be due to the fact that substrates in red leaves are more catalyzed to synthesize downstream products. The “Red” group exhibited significantly higher levels of downstream metabolites, including naringin, delphinidin derivatives, and cyanidin derivatives, than the “Green” group. This result is consistent with its appearance. Furthermore, we also found that in the colored leaves of *Heuchera micrantha*, anthocyanins were mainly composed of delphinidin and cyanidin and their derivatives, while no other types of anthocyanins were identified.

Besides, in flavonoid metabolism, in addition to the biosynthesis of anthocyanins, flavonol synthase and flavonol synthase also catalyze the biosynthesis of kaempferol and luteolin, respectively, which are branch pathways of flavonoid metabolism, and its products are also related to plant coloration (Wang et al., 2021). We analyzed its metabolites and found that the content of kaempferol was significantly higher in the “Green” group, while the “Red” group contained more luteolin (**Figure 3B**). Based on the above results, it preliminarily indicates that the coloration of different colored leaves of *Heuchera micrantha* is mainly influenced by the anthocyanin biosynthesis in the flavonoid metabolism pathway.

### Analysis of transcriptome and identification of differentially expressed genes

**Figure 4:**
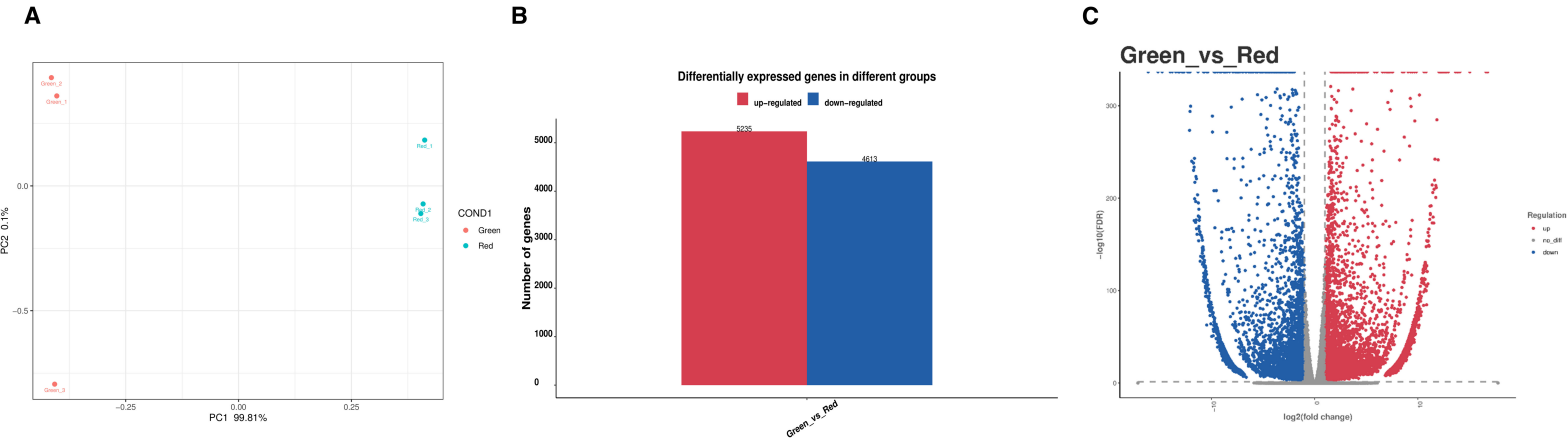
Overview of the transcriptome of *Heuchera micrantha* leaves with different colors. **(A)** The PCA plot shows the distribution of samples with different leaves colors. **(B, C)** The combined analysis of bar chart and volcano chart represents the number of differentially expressed genes (DEGs) in different groups. The red bar and dots represent upregulated genes, while the blue represent downregulated genes. The comparison is Green vs Red.

Six samples were obtained for RNA Seq analysis, with three biological replicates in each of the two colored leaves. The outcome of the assembly procedure was a set of 16753 transcript sequences, with N50 of 2105 bp and GC content of 40.83%, as well as a set of 40863 gene sequences, with N50 of 1848 bp and GC content of 40.84% (**Supplementary Table S1**). PCA showed that PC1 is 99.81% and PC2 0.1%, and the PCA plot showed a significant separation of genetic expression in the “Green” and “Red” groups (**Figure 4A**). Differential expression genes (DEGs) were analyzed by comparing transcriptomes between leaves of different colors (green and red) with thresholds of FDR ≤ 0.05 and |log_2_FC|≥1. In the comparison between “Green” and “Red” groups, there was a significant upregulation of 5235 genes, and a significant downregulation of 4613 genes (**Figures 4B, C**).

### Functional analysis of DEGs

**Figure 5:**
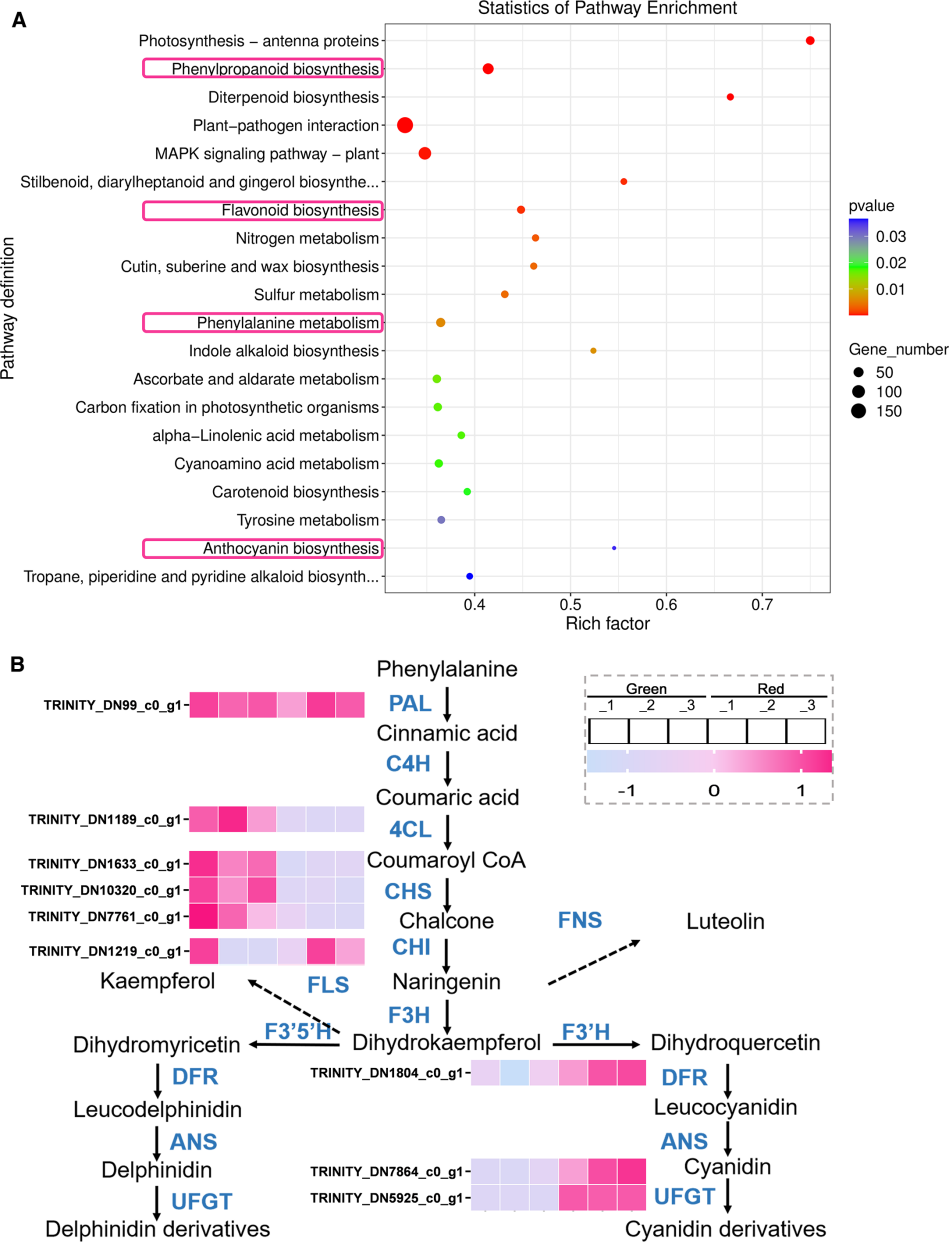
Statistics of pathway enrichment and expression analysis of DEGs. **(A)** KEGG enrichment bubble plots. The size of the dots represents the number of DEGs involved in each pathway, with larger dots indicating higher quantities. The color gradient from blue to red represents the P-value, with red indicating a more significant enrichment. **(B)** DEGs Analysis in different groups of flavonoids biosynthetic pathway. The heatmap displays the expression levels of genes in different populations. The color gradient from purple to pink represents low to high levels of expression. Genes are labeled with their respective Trinity IDs.

Subsequently, in order to further explore the molecular mechanisms behind the differential metabolism between green and red leaves of *Heuchera micrantha*, KEGG enrichment analysis was performed on the DEGs. Consistent with metabolomics analysis, the results showed that the functions of DEGs were mainly enriched in the regulation of metabolic pathways, including Flavonoid biosynthesis, Anthocyanin biosynthesis, Phenylpropanoid biosynthesis, and Phenylalanine metabolism (**Figure 5A**).

Next, we analyzed the expression of biosynthetic genes in Phenylpropanoid metabolism and Flavonoid biosynthesis pathway enriched in the KEGG analysis mentioned above (**Figure 5B**), which will help clarify how the expression of these genes affects metabolite changes and leads to differences in leaf color. As shown in the **Figure 5B**, we found that in the comparative analysis of the DEGs between the “Green” and “Red” groups, there was no significant difference in the expression of the key gene, *PAL* (phenylalanine ammonia lyase, TRINITY_DN99_c0_g1), while *4CL* (4-coumaroyl: CoA ligase, TRINITY-DN1189_c0ug1) in the “Green” group were significantly upregulated, for Phenylpropanoid metabolism. In the subsequent Flavonoid metabolism, the early biosynthetic genes, CHS (chalcone synthase, TRINITY-DN1633_c0ug1, TRINITY-DN10320_c0ug1, TRINITY-DN7761_c0ug1) in the “Green” group were significantly upregulated, while *CHI* (chalcone isomerase, TRINITY-DN1219_c0ug1) showed no significant difference. About the late biosynthetic genes, *DFR* (dihydroflavonol 4-reductase, TRINITY_DN1804_c0_g1) and *UFGT* (UDP-glucose-flavonoid 3-O-glucosyltransferase, TRINITY_DN7864_c0_g1, TRINITY_DN5925_c0_g1) were significantly overexpressed in the “Red” group. The expression trend of the above biosynthetic genes is consistent with the differential accumulation trend of metabolites in the metabolome.

### Characterization of upstream candidate transcription factors from the DEGs analysis

**Figure 6:**
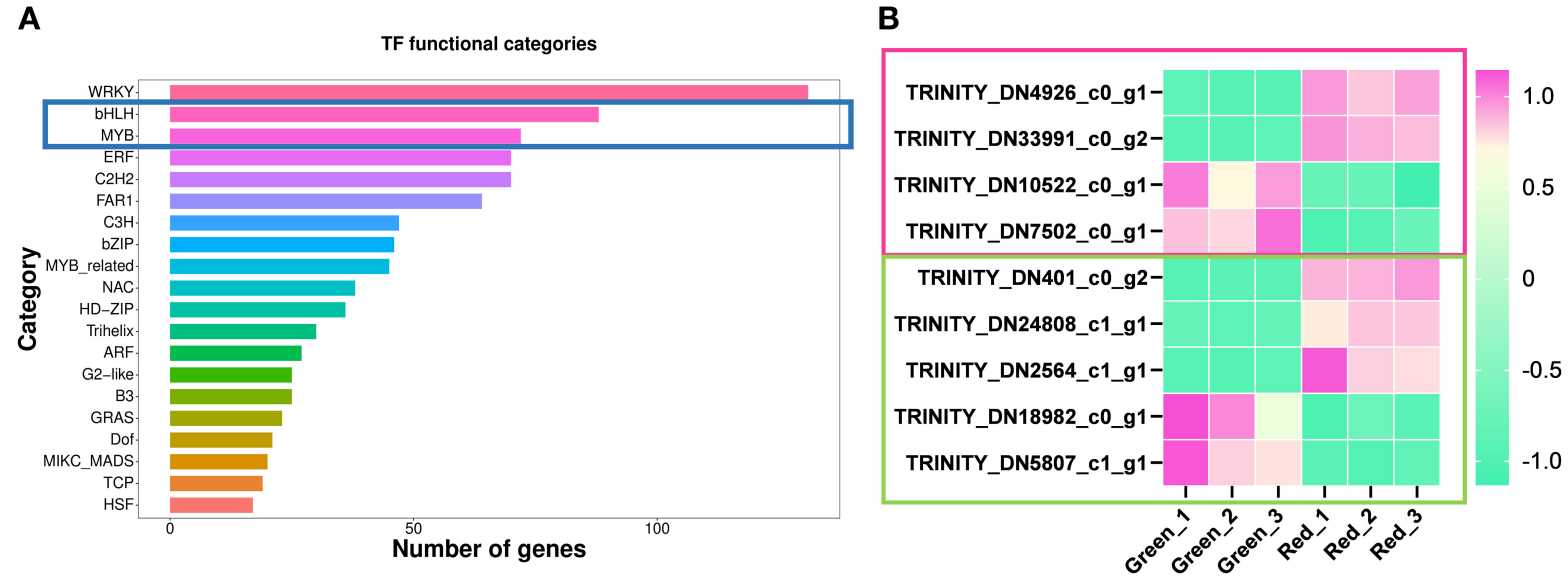
Identification and expression pattern analysis of candidate transcription factors. **(A)** A bar chart displaying the number of genes associated with different transcription factor (TF) functional categories. The categories are listed on the y-axis, and the number of TFs is represented on the x-axis. **(B)** The heatmap shows the expression patterns of MYB and bHLH TFs, which are differentially expressed in green and red comparisons. The color gradient from green to pink represents the level of expression from low to high. TFs are labeled with their respective Trinity IDs, with the pink box representing bHLH TFs and the green box representing MYB TFs.

In plants, flavonoid metabolism is not only regulated by the expression of biosynthetic genes, but also by upstream transcription factors, mainly including bHLH (Liu et al., 2024), MYB (Pratyusha and Sarada, 2022), and MBW complexes (Koes et al., 2005; Lloyd et al., 2017; Naik et al., 2022; Hong et al., 2023). As shown in the **Figure 6A**, a total of 88 bHLH transcription factors and 72 MYB transcription factors were annotated in transcriptome analysis (**Figure 6A**). In order to screen the candidate transcription factors located upstream of flavonoid metabolism pathway, especially candidate members of MBW protein complex, the transcription factors shown in **Figure 6B** were obtained in the DEGs analysis between “Green” and “Red”. As a result, we obtained 4 differentially expressed bHLH transcription factors, including 2 upregulated (TRINITY_DN10522_c0_g1, TRINITY_DN7502_c0_g1) and 2 downregulated (TRINITY_DN4926_c0_g1, TRINITY_DN33991_c0_g2), as well as 5 differentially expressed MYB transcription factors, both upregulated (TRINITY_DN18982_c0_g1, TRINITY_DN5807_c1_g1) and downregulated (TRINITY_DN401_c0_g2, TRINITY_DN24808_c1_g1, TRINITY_DN2564_c1_g1) accounted for half (**Figure 6B**).

In order to further investigate the functions of the candidate TFs mentioned above, we conducted motif analysis of the TFs using MEME Suite 5.5.8 (https://meme-suite.org). The results of motif positions and sequences are shown in **Supplementary Figure S1**. As shown in the figure, TFs in the same TF family share common motifs, but they also differ from each other. Next, we performed a blast on the protein sequences of candidate TFs at NCBI (The National Center for Biotechnology Information, https://blast.ncbi.nlm.nih.gov/Blast.cgi) to explore their potential biological functions. About MYB TFs, we found that TRINITY_DN401_c0_g2 had the highest homology with LfMYB113 (AQM49950.1), which was a positive regulator of anthocyanins accumulation in *Liquidambar formosana* leaves (Wen et al., 2015); And TRINITY_DN24808_c1_g1 had the highest homology with a PsMYB (QIG55720.1), which has not yet been characterized; TRINITY_DN2564_c1_g1 had the highest homology with LjMYB12 (QER90717.1), which regulates flavonoid metabolism positively in *Lonicera japonica* (Wang et al., 2019). These results ulteriorly indicate that the above three MYBs positively regulate flavonoid metabolism in *Heuchera micrantha*. The candidate negative regulators of flavonoid metabolism in *Heuchera micrantha*, TRINITY_DN18982_c0_g1 and TRINITY_DN5807_c1_g1, had the highest homology with *Phaseolus vulgaris* PvMYB114-like (XP_031247895.1) and *Telopea* sp*eciosissima* TsMYB1-like (XP_043722769.1), respectively.

As for the bHLH TFs, the results showed that TRINITY_DN4926_c0_g1 and TRINITY_DN33991_c0_g2 had highly similar motifs (**Supplementary Figure S1**), and both have the highest homology with VvbHLH87 (RVX17028.1) in blast, which indicated that the two may be different copies or variable splicing of the same gene. Although the function of VvbHLH87 had not been characterized, the expression trend of the above candidate bHLHs suggested that they may be positive regulators of flavonoid biosynthesis. And the potential negative regulatory TFs of flavonoid metabolism, TRINITY_DN10522_c0_g1 and TRINITY_DN7502_c0_g1, had the highest homology with *Vitis vinifera* VvbHLH51 (RVX03143.1) and *Cunninghamia fargesii* CfbHLH62-like (XP_059632074.1), respectively.

### Combined analysis of transcriptome and metabolome to explore the formation mechanism of different leaf colors

**Figure 7:**
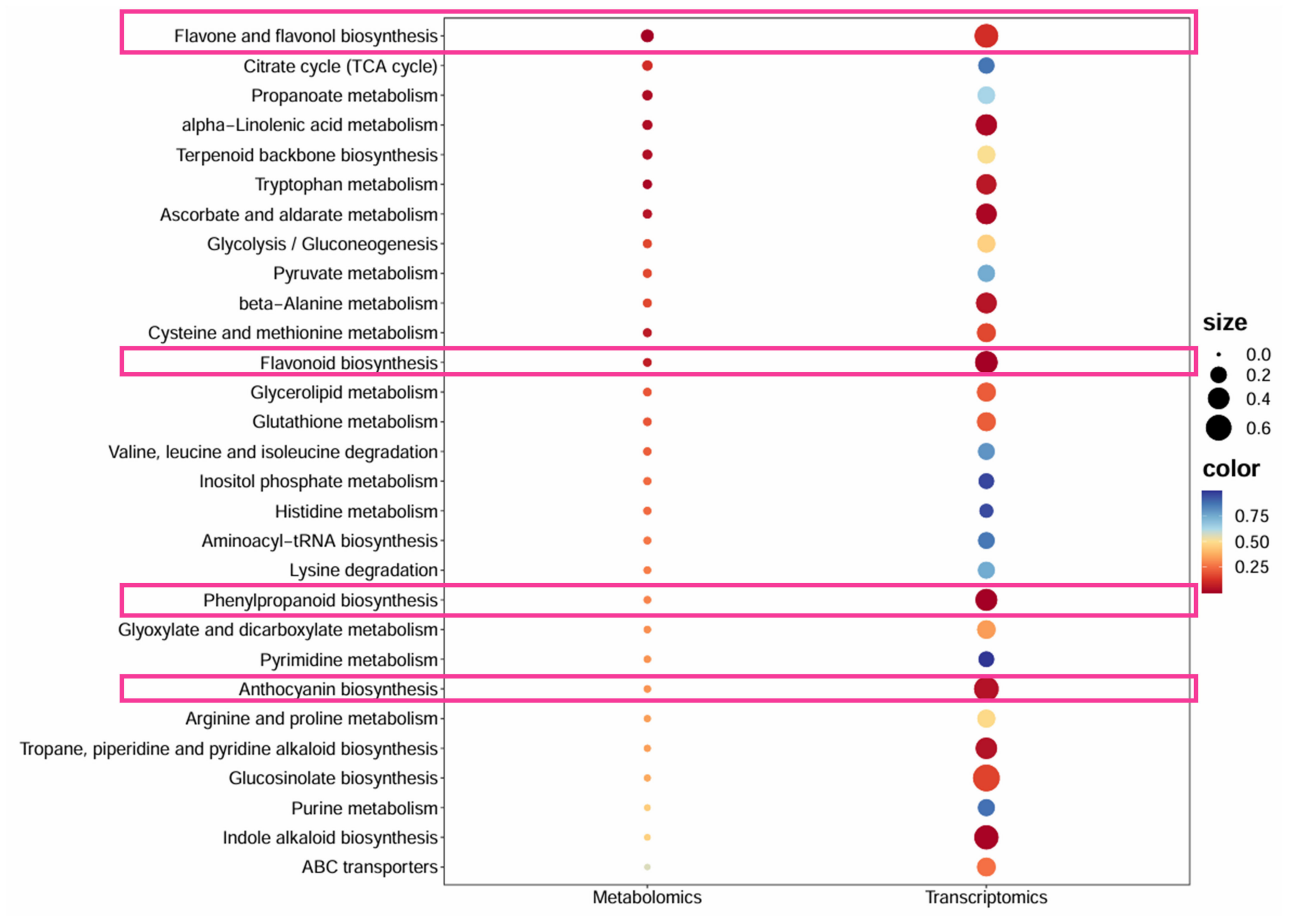
The KEGG bubble plot of the common pathway between transcriptome and metabolome in different color leaves. The x-axis lists metabolic pathways, with P-values represented by a gradient from blue to red, where red indicates more significant enrichment (lower P-values), and the size of each bubble corresponds to the degree of enrichment. The pink box represents flavonoid metabolism and its related metabolic pathways.

In order to overcome the problem that it is difficult to associate genes with phenotypes in a single omic analysis, we conducted a joint analysis of transcriptome and metabolome of *Heuchera micrantha* with different leaf colors. We screened and obtained pathways that were significantly different in both metabolomics and transcriptomics, as shown in **Figure 7**. In the joint analysis of red and green leaves, we identified that the metabolism of flavonoid and flavonol biosynthesis, flavonoid biosynthesis, phenylpropane biosynthesis and anthocyanin biosynthesis were significantly enriched in the above two omics, as shown in the pink box in **Figure 7**. Besides, we found that structural genes in red leaves were significantly upregulated at the branching nodes of flavonoid metabolism (**Figure 5B**), while the significant high accumulation of metabolites was reflected in naringenin and the final products of the flavonoid biosynthesis (**Figure 3B**), which may be due to the lag between metabolite accumulation and gene expression. The above results further showed that the metabolite difference of phenylpropane to flavonoid metabolism was an important reason for the formation of color-leafed, which was regulated by the differential expression of structural genes related to this pathway and upstream candidate transcription factors. This result provides a genetic reserve for subsequentcolor-leafed directional breeding. In a word, this preliminary revealed the formation mechanism of different leaf colors of *Heuchera micrantha*, and provided genetic reserves for subsequent oriented breeding.

## Discussion

In this study, we systematically analyzed the molecular mechanisms underlying the differences in leaf color between red and green leaves of *Heuchera micrantha* by integrating metabolomics and transcriptomics. Research has found that the flavonoid metabolism pathway, especially the anthocyanin biosynthesis pathway, is the core pathway driving differences of leaf colors. Metabolome data showed that delphinidin, cyanidin, and their derivatives were significantly enriched in red leaves, while upstream substrates such as phenylalanine and cinnamic acid accumulated more in green leaves (**Figure 3**). Transcriptome analysis further confirmed the molecular mechanism behind the above conclusion, that is, *DFR* and *UFGT* (late synthesis gene) were significantly upregulated in red leaves, while *CHS* (early synthesis gene) was expressed higher in green leaves (**Figure 5**). These findings match the leaf-color phenotype (**Figure 1**) and parallel earlier work in kale (Zhu et al., 2018), red coloration coincides with the accumulation of late anthocyanin products, whereas green leaves may exhibit a low anthocyanin state due to substrate diversion to other branches (such as flavonol biosynthesis), which is similar to the results of some previous studies on competitive catalysis of substrates in flavonoid metabolism, such as chrysanthemum, dahlia, and rose (Deguchi et al., 2013; Luo et al., 2015; Wang et al., 2021). It is worth noting that only two types of anthocyanins, delphinidin, cyanidin, and their derivatives were detected in *Heuchera micrantha*, indicating that their biosynthesis pathways are species-specific and may be related to substrate preferences of Flavonoid 3’,5’-hydroxylase (F3’5’H) and DFR enzymes (Katsumoto et al., 2007; Noda et al., 2017). Integrating transcriptome and metabolome data solidifies these conclusions.

Subsequently, we identified several differentially expressed MYB and bHLH transcription factors (**Figure 6**), which may be individually involved in flavonoid metabolism regulation or regulate anthocyanin biosynthesis through the formation of MBW complexes. No differentially expressed WD40 was detected, consistent with previous reports that MYB–bHLH complexes can activate anthocyanin genes without WD40 participation (Liu et al., 2013; Lai et al., 2016; Sun-Hyung et al., 2017). Further protein sequence analysis revealed that bHLH and MYB transcription factors share common conserved motifs, respectively, but within the same family, their motifs also differ, suggesting that they may belong to different subfamilies and play different biological functions (**Supplementary Figure S1**). DEGs analysis of candidate TFs suggested that 2 bHLH TFs and 3 MYB TFs, namely TRINITY_DN4926_c0_g1, TRINITY_DN33991_c0_g2TRINITY_DN401_c0_g2, TRINITY_DN24808_c1_g1, and TRINITY_DN2564_c1_g1, may be positive regulators of anthocyanin biosynthesis, while 2 bHLH TFs and 2 MYB TFs, TRINITY_DN10522_c0_g1, TRINITY_DN7502_c0_g1, TRINITY_DN18982_c0_g1, and TRINITY_DN5807_c1_g1, have the opposite function (**Figure 6**). However, their clearly biological function and regulatory mechanism still need to be verified in subsequent transgenic experiments.

*Heuchera mirantha* is an excellent germplasm of color-leafed plants in cold regions, the study on the formation mechanism of different leaf colors not only provides key gene resources for molecular breeding, but also provides a certain reference for the research of other color-leafed plants. Moreover, *Heuchera mirantha* has more leaf colors besides the red and green, and even has compound colored leaves. Representative red and green leaves varities were selected in the study, and single colored leaves can better eliminate cross pathways to preliminarily elucidate the coloring mechanism. The metabolic mechanisms of other plant pigments involved, such as carotenoids, chlorophyll and etc., still need to be explored. For example, in our differential metabolite analysis, we identified that geranylgeranyl chlorophyll-a was significantly higher in green leaves than in red. And the regulation of transcription factors and their response to environmental factors are also important research directions for the future.

## Data Availability

The datasets presented in this study can be found in online repositories. The names of the repository/repositories and accession numbers can be found in the article/**Supplementary Material**.

## Supplementary material

Supplementary Figure 1 The motif analysis of candidate transcriptional factors using MEME suite 5.5.8, with pink boxes representing bHLH transcription factors and green boxes representing MYB transcription factors.

Supplementary Table 1 Summary of transcriptome sequencing data for *Heuchera micrantha* leaves with two different colors.

Supplementary Table 2 The details about the R package used for data analysis.
