# Supplementary figures and images for: Metabolomics combined with transcriptomics reveals the formation mechanism of different leaf colors of Heuchera micrantha
Source: Front Plant Sci. 2025 Sep 26;16:1672924. doi: 10.3389/fpls.2025.1672924 (PMC12511031; doi:10.3389/fpls.2025.1672924)

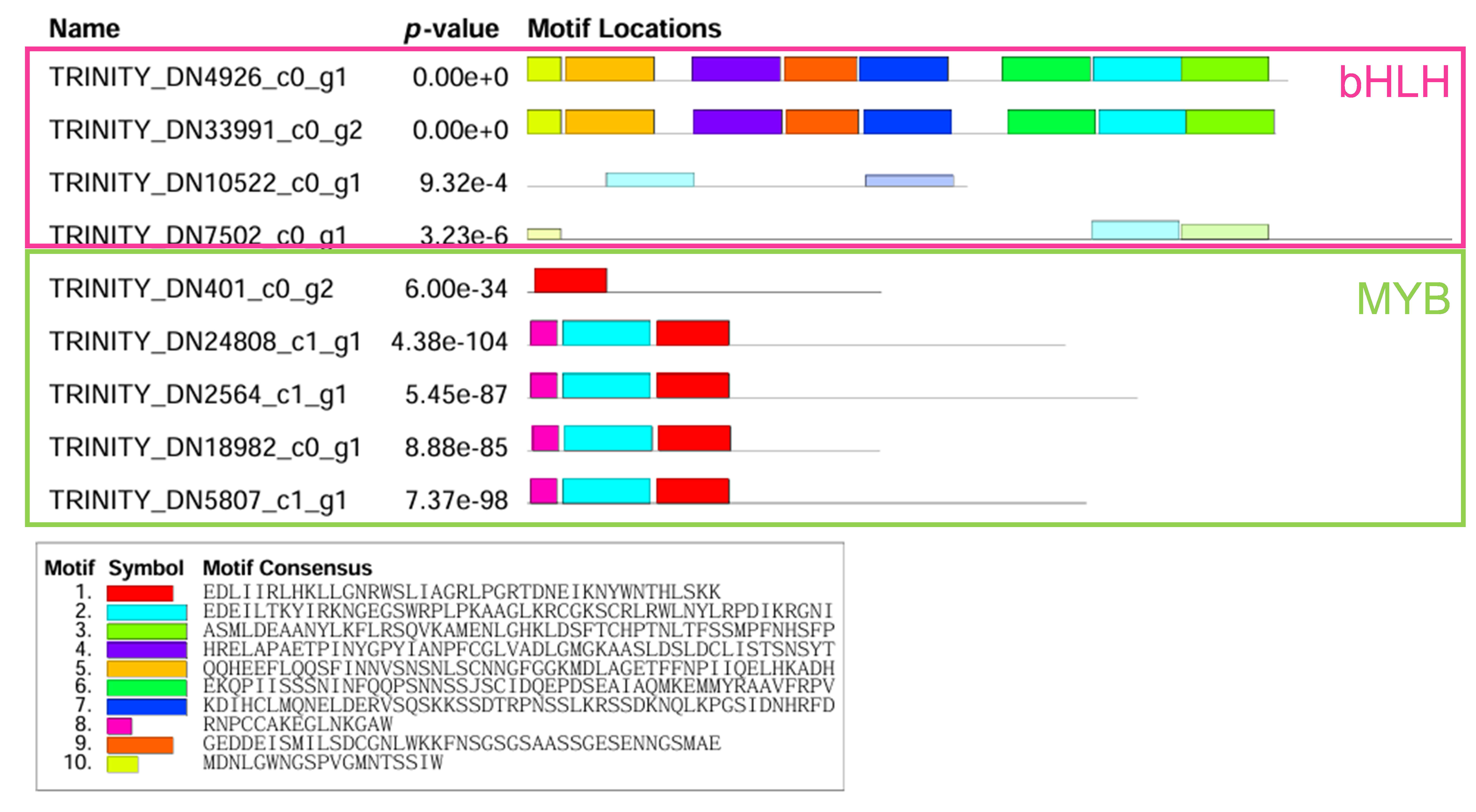

Supplement: Supplementary Figure 1 — The motif analysis of candidate transcriptional factors using MEME suite 5.5.8, with pink boxes representing bHLH transcription factors and green boxes representing MYB transcription factors. [file Image1.tif]
